# National Early Warning Score 2 Lactate (NEWS2-L) in Predicting Early Clinical Deterioration in Patients with Dyspnoea in Prehospital Care

**DOI:** 10.17533/udea.iee.v39n3e05

**Published:** 2021-11-05

**Authors:** Raúl Villanueva-Rábano, Francisco Martín-Rodríguez, Raúl López-Izquierdo

**Affiliations:** 1 Nurse, M.Sc. Intensive Care Medicine Department, University Clinical Hospital of Valladolid Spain Email: raulderivia@gmail.com. Corresponding author. University Clinical Hospital of Valladolid Spain raulderivia@gmail.com; 2 Nurse, Ph.D. Valladolid I Emergency Mobile Unit, Health Emergencies Management. Spain Email: fmartin@saludcastillayleon.es. Valladolid I Emergency Mobile Unit Spain fmartin@saludcastillayleon.es; 3 Physician, Ph.D. Emergency Department, Rio Hortega University Hospital of Valladolid. Spain Email: rlopeziz@saludcastillayleon.es. Rio Hortega University Hospital of Valladolid Spain rlopeziz@saludcastillayleon.es; 4 Castilla y León Regional Health Management (SACYL), Spain. Spain; 5 Advanced Clinical Simulation Centre, Department of Medicine, Dermatology and Toxicology, University of Valladolid, Spain. Universidad de Valladolid Department of Medicine, Dermatology and Toxicology University of Valladolid Spain

**Keywords:** dypsnea, biomarkers, prehospital care, early warning score, hospital mortality, clinical decision-making., disnea, biomarcadores, atención prehospitalaria, puntuación de alerta temprana, mortalidad hospitalaria, toma de decisiones clínicas, dispneia, biomarcadores, assistência pré-hospitalar, escore de alerta precoce, mortalidade hospitalar, tomada de decisão clínica.

## Abstract

**Objective:**

To evaluate the ability of the NEWS2-L (National Early Warning Score 2 Lactate) scale to predict the risk of early clinical deterioration (mortality within 48 hours) in patients with dyspnoea treated by the Medical Emergency Services compared with NEWS2 and lactate in isolation.

**Methods:**

Prospective, multi-centre study of a cohort of 638 patients with dyspnoea treated in the ambulance and priority-transferred to a hospital emergency service in the cities of Valladolid, Salamanca, Segovia or Burgos (Spain). We collected clinical, analytical and demographic data. The main outcome measure was all-cause mortality within 48 hours. The recommendations of the Royal College of Physicians were followed to calculate NEWS2. When NEWS2 and LA prehospital values were obtained, the two values were added together to obtain the NEWS2-L.

**Results:**

Mortality within 48 hours was fifty-six patients (8.8%). The NEWS2-L scale obtained an area under the curve (AUC) of the receiver operating characteristics (ROC) for mortality within 48 hours of 0.854 (CI 95% 0.790-0.917), at seven days of 0.788 (CI 95% 0.729-0.848) and at 30 days of 0.744 (CI 95% 0.692-0.796); in all cases *p*<0.001, with a significant decrease between the value at 48 hours and at 30 days.

**Conclusion:**

The NEWS2-L scale was found to be significantly superior to the NEWS2 scale and similar to lactate in predicting early clinical deterioration in patients with dyspnoea. This scale can help a nurse detect these patients early, as part of their regular practice, and thus guide therapeutic efforts.

## Resumen

**Objetivo:**

Evaluar la capacidad de la escala NEWS2-L (National Early Warning Score 2-lactate) para predecir el riesgo de deterioro clínico precoz (mortalidad hasta las 48h) en pacientes con disnea atendidos por Servicios de Emergencias Médicas, comparado con la escala NEWS2 y el ácido láctico en solitario**.**

**Métodos:**

Estudio prospectivo multicéntrico de cohorte de 638 pacientes con disnea atendidos en ambulancia y trasladados con alta prioridad a un servicio de urgencias hospitalarias en las ciudades de Valladolid, Salamanca, Segovia y Burgos (España). Se tomó información de variables clínicas, analíticas y demográficas, de las cuales la de resultado principal fue la mortalidad por cualquier causa hasta las 48 horas. Para el cálculo del NEWS2 se siguieron las recomendaciones del Royal College of Physicians. Una vez obtenidos los valores del NEWS2 y del AL prehospitalario se sumaron ambos valores y se obtuvo la NEWS2-L.

**Resultados:**

La mortalidad, antes de las 48, horas fue de 56 pacientes (8.8%). La escala NEWS2-L obtuvo un Área Bajo la Curva - Característica Operativa del Receptor (ABC-COR) para la mortalidad antes de las 48 horas de 0.854 (IC95% 0.790-0.917), a siete días de 0.788 (IC95% 0.729-0.848) y a 30 días de 0.744 (IC95% 0.692-0.796); en todos los casos *p*<0.001), lo que experimentó un descenso importante entre su valor a las 48 h y a los 30 días.

**Conclusión:**

La escala NEWS2-L mostró ser significativamente superior a la escala NEWS2 y similar al ácido láctico en la predicción del deterioro clínico precoz en pacientes con disnea. Esta escala es una ayuda para que la enfermera en su práctica habitual detecte a estos pacientes en forma temprana y así poder orientar los esfuerzos terapéuticos.

## Resumo

**Objetivo:**

Avaliar a capacidade da escala NEWS2-L (National Early Warning Score 2-lactato) de predizer o risco de deterioração clínica precoce (mortalidade de até 48h) em pacientes com dispneia tratados em Serviços de Emergência Médica, em comparação com a escala NEWS2 e a ácido láctico em solitário.

**Métodos:**

Estudo prospectivo de coorte multicêntrico de 638 pacientes com dispneia atendidos por ambulância e transferidos com alta prioridade para um serviço de emergência hospitalar nas cidades de Valladolid, Salamanca, Segovia e Burgos (Espanha). As informações foram obtidas a partir de variáveis ​​clínicas, analíticas e demográficas, sendo a principal variável de desfecho a mortalidade por todas as causas em até 48 horas. Para o cálculo do NEWS2, foram seguidas as recomendações do Royal College of Physicians. Uma vez obtidos os valores do NEWS2 e do AL pré-hospitalar, ambos os valores foram somados e o NEWS2-L foi obtido.

**Resultados:**

A mortalidade antes de 48 horas foi de 56 pacientes (8,8%). A escala NEWS2-L obteve uma área sob a curva - característica operacional do receptor (ABC-COR) para mortalidade antes de 48 horas de 0.854 (IC 95% 0.790-0.917), em sete dias de 0.788 (IC 95% 0.729-0.848) e aos 30 dias de 0.744 (95% CI 0.692-0.796); em todos os casos p <0,001), experimentando uma diminuição significativa entre o seu valor às 48 he aos 30 dias.

**Conclusão:**

A escala NEWS2-L mostrou ser significativamente superior à escala NEWS2 e semelhante ao ácido láctico na predição da deterioração clínica precoce em pacientes com dispneia. Essa escala é um auxílio para o enfermeiro em sua prática habitual detectar precocemente esses pacientes e, assim, ser capaz de orientar os esforços terapêuticos.

## Introduction

Dyspnoea is defined as a subjective feeling of a lack of air or difficulty breathing and is a symptom present in a great variety of pathologies, often associated with different degrees of respiratory failure.(1) Dyspnoea represents nearly 50% of patients admitted to Tier 3 hospitals, with the figure falling to around 25% in outpatient centres.(2) It also accounted for 3.7 million annual visits to emergency departments in the United States.(3) Dyspnoea is also present in a large number of patients admitted to intensive care units (ICUs),(4) consuming a large quantity of health system resources.

At the prehospital level, it is a common reason to seek medical care, with different levels of mortality and morbidity.^5^ These patients often have to be treated by prehospital emergency services and many require evacuation from the site in high-priority situations. As mentioned, dyspnoea is a symptom that in addition to being an independent predictor of mortality in many clinical situations is associated with a complex and heterogeneous group of patients that present different serious diseases with multiple co-morbidities, involving a wide range of diagnostic possibilities including heart failure, chronic obstructive pulmonary disease (COPD) and respiratory tract infections.(6,7)

Given the clinical complexity patients with dyspnoea present and considering the importance of a rapid detection of deterioration in these patients, it is important to find which tool is optimal in establishing a prognosis for them from the first moments of care. In this context, the National Early Warning Score 2 (NEWS2) is one of the scales validated in the prehospital setting and has a correlation between a high prehospital score in NEWS2 and a higher incidence of adverse outcomes.^8^^-^10^)^ The role of lactate (LA) as a predictor of poor prognosis generally in patients treated by mobile and hospital emergency services is also well known,(11) acting as a classic anaerobic metabolism marker in the body, indicating hypoxia and tissue hypoperfusion. (12)

The detection and handling of early signs of deterioration in the patient is a highly complex process influenced by many factors. In this regard, nursing personnel play a very important role in identifying clinical deterioration in potentially critical patients due to how closely they work with the patient. Understanding and handling early warning scales like NEWS2 and the meaning of biomarkers like LA is particularly important in these circumstances and can serve as an aid for nurses to guide their actions.(13) The aim of this study was to assess the ability of the NEWS2-L (National Early Warning Score 2 Lactate) scale to predict the risk of early clinical deterioration (mortality within 48 hours) in patients with dyspnoea treated by the medical emergency services compared with the NEWS2 scale and lactate in isolation. A secondary goal was to check the scale’s prognostic ability to determine mortality at seven and 30 days.

## Methods

Study design and ethical considerations. We conducted a prospective, multi-centre cohort study on an opportunity sample of patients with dyspnoea treated by an Emergency Mobile Unit (EMU) in the cities of Valladolid, Salamanca, Segovia and Burgos (Spain). These patients were subsequently transferred to their reference hospital. The main outcome measure was all-cause mortality within 48 hours. Secondarily, mortality at seven and 30 days after treatment by the EMU was studied at a global level.

Participants. Between 1 April 2018 and 30 June 2019, patients aged ≥ 18 years were included if they had called the 112 emergency number requesting urgent help and after assessment by the ambulance (EMU) on the scene were determined to have dyspnoea (medical origin) as the reason for their call and were taken to their reference hospital by ambulance. Excluded from the study were patients where it was not possible to obtain informed consent, patients under the age of 18, pregnant women, patients with an acute psychiatric disorder or documented terminal disease, patients who died during the callout or transfer, patients evacuated by other means of transport (e.g., basic life support units or private means) or patients who following assessment by the EMU required no further urgent care and were discharged onsite.

Variables and data collection. The endpoints and predictors were collected by independent researchers at each hospital, obtained from a review of patient electronic medical records. The primary endpoint was collected by the clinical investigators tasked with data collection. Epidemiological values were also collected during the callout (age, sex, arrival times, treatment and transfer and reason for the call), as were the clinical values needed to calculate NEWS2 (respiratory rate, oxygen saturation, use of supplemental oxygen, systolic blood pressure, heart rate, temperature and level of consciousness; confusion was considered a score on the Glasgow Coma Scale of under 15 points). Analytical values (glucose and venous LA) were also collected. The prehospital main diagnosis was also recorded on the basis of the International Classification of Diseases (ICD-11, https://icd.who.int/browse11/l-m/en). The heart rate, blood pressure and oxygen saturation measurements were performed with the LifePak® 15 monitor (Physio-Control, Inc., Redmond, USA). Temperature was obtained with a tympanic thermometer, model ThermoScan® PRO 6000 (Welch Allyn, Inc, Skaneateles Falls, USA), glucose was obtained with the FreeStyleOptium Neo glucometer (Abbott Laboratories, Illinois, USA) and LA with the Accutrend Plus lactometer (Roche Diagnostics, Mannheim, Germany).

By reviewing the patient’s electronic medical record 30 days after the index event, the following hospital values were recorded: admission, need for ICU, in-patient days and mortality at 48 hours and at seven and 30 days.

NEWS2-L determination. The recommendations of the Royal College of Physicians(14) were followed to calculate NEWS2 (see Table 1).

**Table 1:** National Early Warning Score 2 Scale

NEWS2	3	2	1	0	1	2	3
HR (bpm)	≤ 40		41-50	51-90	91-110	111-130	≥131
RR (bpm)	≤8		9-11	12-20		21-24	≥25
T (ºC)	≤35		35.1-36	36.1-38	38.1-39	≥39.1	
SBP (mmHg)	≤90	91-100	101-110	111-219			≥220
SpO2 (%) Scale 1	≤91	92-93	94-95	≥96			
SpO2 (%) Scale 2	≤83	84-85	86-87	88-92 ≥93 Air	93-94 Oxygen	95-96 Oxygen	≥97 Oxygen
Suppl. O2		Oxygen		Air			
GCS (points)				15			≤14

Scale 2 should be used in patients with hypercapnic respiratory failure to weigh up the oxygen saturation score. Each category was classified from zero to three points. The scores of each category were added together to obtain a total. Composite scores over five (or three in any of the parameters) triggered an urgent review. A score over seven triggered a review by the critical care team or an advanced medical response team. Abbreviations: NEWS2: National early warning score 2; HR: heart rate; RR: respiratory rate; T: temperature; SBP: systolic blood pressure; SpO2: oxygen saturation; suppl. O2: supplemental oxygen; GCS: Glasgow coma scale. Taken from the Royal College of Physicians.(14)

The LA values were collected by the nursing personnel of each EMU in three phases: 1) The test strip was inserted after switching on the instrument. 2) A drop of venous blood (15-40 μL) was deposited on the test strip. 3) The lid was closed and a result obtained after 60 seconds. The maximum time between blood collection and placement of the sample in the device was one minute. All the measuring devices were calibrated every 100 measurements, always by the same researcher, using the Accutrend® BM-Control-Lactate control solution (Roche Diagnostics, Mannheim, Germany). When the NEWS2 and LA prehospital values were obtained, the two values were added together to obtain NEWS2-L.

Statistical analysis. All the data was stored in an XLSTAT® BioMED database for Microsoft Excel® version 14.4.0. (Microsoft Inc., Redmond, WA. USA), and SPSS version 20.0. (IBM, Armonk, NY. USA) which were also used for statistical analysis. The database was cleaned prior to statistical analysis by means of logical tests, range tests (for the detection of extreme values) and data consistency. The presence and distribution of unknown values of all variables were subsequently analysed. Qualitative variables were described by absolute frequencies with their 95% confidence interval (CI 95%). Quantitative variables were described as median and interquartile range (IQR). The Mann-Whitney U-test was used to compare quantitative variables. The Chi-square test for two-way tables or the contrast of proportions was used to determine the association or dependence relationship between qualitative variables; if necessary (percentage of boxes with expected values less than five, greater than 20%), we used Fisher’s exact test. NEWS2, NEWS2-L and LA area under the curve (AUC) of the receiver operating characteristic (ROC) was calculated for mortality at 48 hours and at seven and 30 days, along with the best score in each case for greatest combined sensitivity and specificity (Youden index). We also calculated the positive predictive value (PPV), negative predictive value (NPV), positive probability ratio (PPR) and negative probability ratio (NPR) for these scores. In all tests, a confidence level of 95% and a p-value of less than 0.05 were considered significant. The values obtained were subsequently compared against each other, establishing a comparison between the three tests studied (NEWS2-L vs NEWS2 vs LA).

Ethical aspects. The study was approved by the Clinical Research Ethics Committees of all the participating centres. All the patients (or guardians) signed the informed consent form. The highest safety standards were followed at all times, protecting participant confidentiality and complying with the national and international regulations included in the Declaration of Helsinki on biomedical research involving human subjects.

## Results

**Figure 1:**
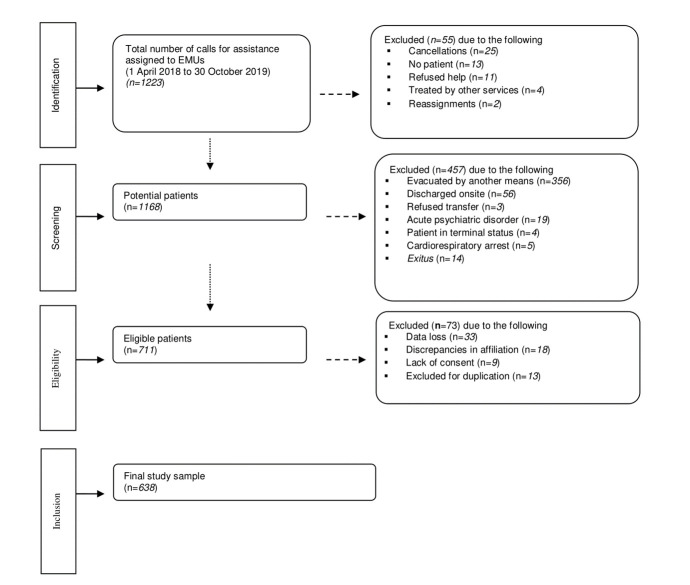
Diagram of participants

A total of 638 patients with dyspnoea were included between 1 April 2018 and 30 October 2019 (Figure 1). Mortality at two days was 8.8% (56 patients). Median age was 79 years (IQR 68-86 years), with 39.9% women (255 patients).

Stratifying the sample by predominant pathology in patients with dyspnoea who were treated and transferred, 170 (26.6%) were patients with chronic obstructive pulmonary disease (COPD), 164 (25.7%) were patients with heart failure (HF), 198 (31%) were patients with infections and the remaining 106 (16.6%) were patients with any condition other than the above, covered under the mixed group of ‘Other’. The values of the patients’ clinical/epidemiological characteristics were provided as absolute and percentage values or as median and interquartile range as appropriate. Abbreviations: IQR: interquartile range; RR: respiratory rate; SpO2: oxygen saturation; O2: oxygen; SBP: systolic blood pressure; HR: heart rate; GCS: Glasgow coma scale; LA: lactate; NEWS2: National Early Warning Score 2; NEWS2-L: National Early Warning Score 2 Lactate; COPD: chronic obstructive pulmonary disease; HF: heart failure; ICU: intensive care unit (Table 2).

**Table 2:** Clinical-epidemiological characteristics of participants. The mortality statistics refer to death within two days**.**

Characteristics	Total	Mortality at 48 hours	Mortality at 48 hours	***p_*value**
Characteristics	Total	Survivors	Non-survivors	***p_*value**
Number; n (%)	638	582 (91.2)	56 (8.8)	
Sex: female: (n (%)	255 (39.9)	239 (41.1)	16 (28.6)	0.068
Age (years); Median (IQR)	79 (68-86)	78 (68-85)	81 (72-88)	0.09
Time (minutes); Median (IQR)				
Arrival	10 (8-13)	10 (8-13)	10 (8-14)	0.681
Assistance	30 (25-36)	30 (24-36)	31 (26-39)	0.830
Transfer	9 (7-13)	10 (7-12)	9 (7-17)	0,122
Prehospital assessment; Median (IQR)				
RR (bpm)	26 (19-35)	26 (19-34)	34 (24-38)	0.028
SpO2 (%)	89 (80-94)	90 (82-95)	78 (67-84)	<0.001
O2; (n (%)	256 (40.1)	228 (39.2)	28 (50)	0.115
SBP (mmHg)	140 (121-164)	142 (123-165)	132 (98-151)	0.021
HR (bpm)	100 (80-115)	99 (80-115)	103 (90-125)	0.055
Temperature (ºC)	36.7 (36-37.5)	36.7 (36-37.5)	36.6 (35.5-37.4)	0.064
GCS (points)	15 (15-15)	15 (15-15)	11 (6-15)	<0.001
Blood sugar (mg/dl)	139 (115-180)	138 (115-178)	150 (124-209)	0.058
LA (mmol/L)	3.1 (2.1-4.1)	2.9 (2-3.8)	4.9 (4.3-6.9)	<0.001
Scales: Median (IQR)				
NEWS2	8 (5-10)	7 (5-9)	11 (9-14)	<0.001
NEWS2-L	10.7 (7.5-13.7)	10.3 (7.2-12.8)	17.1 (13.5-20)	<0.001
In-patient follow up; n (%)				
O2	428 (67)	374 (64.3)	54 (96.4)	<0.001
Admitted	505 (79)	450 (77.3)	56 (100)	<0.001
ICU	75 (11.7)	57 (9.8)	18 (32.1)	<0.001
Diagnostic groups; n (%)				
COPD	170 (26.6)	162 (27.8)	8 (14.3)	0.063
HF	164 (25.7)	149 (25.6)	15 (26.8)	
Infections	198 (31)	175 (30.1)	23 (41.1)	
Other	106 (16.6)	96 (16.1)	10 (17.9)	

Values provided as absolute and percentage values or as median and interquartile range as appropriate. Abbreviations: IQR: interquartile range; RR: respiratory rate; SpO2: oxygen saturation; O2: oxygen; SBP: systolic blood pressure; HR: heart rate; GCS: Glasgow coma scale; LA: lactate; NEWS2: National Early Warning Score 2; NEWS2-L: National Early Warning Score 2 Lactate; COPD: chronic obstructive pulmonary disease; HF: heart failure; ICU: intensive care unit.

Comparing the NEWS2 scale vs LA vs NEWS2-L, the NEWS2-L scale obtained the best AUC-ROC for mortality at 48 hours with 0.854 (CI 95% 0.790-0.917; p<0.001) with a cut-off point of 12.2. Similarly, the NEWS2-L scale exceeded the other two tests for seven and 30 days, with AUC-ROCs for the NEWS2-L scale of 0.788 (CI 95% 0.729-0.848; p<0.001) and 0.744 (CI 95% 0.692-0.796; p<0.001) and cut-off points of 12.7 and 12.3, respectively. Comparing the three tests, lactate obtained the best sensitivity (91.1%) and specificity (79.4%) in mortality at 48 hours, followed by the NEWS2-L scale and with a considerable difference with respect to the NEWS2 scale (Table 3).

**Table 3:** Best-scoring combined sensitivity and specificity cut-off points (Youden index) for mortality at 48 hours and at seven and 30 days for NEWS2, NEWS2-L and LA

Mortality	Mortality	Mortality	Mortality
	48 hours	7 days	30 days
Number; n (%)	56 (8.8)	84 (13)	130 (20)
NEWS2	NEWS2	NEWS2	NEWS2
Median (IQR)	8 (5-10)	8 (5-10)	8 (5-10)
AUC (CI 95%)	0.809 (0.739-0.879)	0.755 (0.692-0.817)	0.715 (0.662-0.769)
p value	<0.001	<0.001	<0.001
Cut-off point	10	11	9
Se (CI 95%)	71.4 (58.5-81.6)	53.6 (43.0-63.8)	66.9 (58.5-74.4)
Sp (CI 95%)	75.8 (72.1-79.1)	85.7 (82.6-88.4)	66.9 (62.7-70.9)
PPV (CI 95%)	22.1 (16.7-28.7)	36.3 (28.4-45.0)	34.1 (28.6-40.1)
NPV (IC 95%)	96.5 (94.4-97.8)	92.4 (89.8-94.4)	88.8 (85.2-91.6)
PPR (CI 95%)	2.95 (2.37-2.67)	3.76 (2.82-5.0)	2.02 (1.70-2.41)
NPR (CI 95%)	0.38 (0.25-0.58)	0.54 (0.43-0.69)	0.49 (0.38-0.64)
Diagnostic accuracy	75.4 (71.9-78.6)	81.5 (78.3-84.3)	66.9 (63.2-70.5)
Odds ratio	7.82 (4.25-14.39)	6.94 (4.25-11.33)	4.09 (2.72-6.17)
NEWS2-L	NEWS2-L	NEWS2-L	NEWS2-L
Median (IQR)	10.7 (7.57-13.7)	10.7 (7.57-13.7)	10.7 (7.57-13.7)
AUC (CI 95%)	0.854 (0.79-0.917)	0.788 (0.729-0.848)	0.744 (0.692-0.796)
p value	<0.001	<0.001	<0.001
Cut-off point	12.2	12.7	12.3
Se (CI 95%)	87.5 (76.4-93.8)	76.2 (66.1-84.0)	66.2 (57.7-73.7)
Sp (CI 95%)	70.3 (66.4-73.8)	75.5 (71.7-78.9)	73.8 (69.8-77.5)
PPV (CI 95%)	22.1 (17.1-28)	32.0 (25.9-38.8)	39.3 (33.0-45.9)
NPV (CI 95%)	98.3 (96.6-99.2)	95.4 (93.1-97.0)	89.5 (86.2-92.1)
PPR (CI 95%)	2.94 (2.51-3.45)	3.10 (2.57-3.75)	2.53 (2.09-3.06)
NPR (CI 95%)	0.18 (0.09-0.36)	0.32 (0.21-0.47)	0.46 (0.36-0.59)
Diagnostic accuracy	71.8 (68.2-75.1)	75.5 (72.1-78.7)	72.3 (68.7-75.6)
Odds ratio	16.55 (7.35-37.26)	9.84 (5.74-16.85)	5.51 (3.64-8.33)
Lactate	Lactate	Lactate	Lactate
Median (IQR)	3 (2-4.1)	3 (2-4.1)	3 (2-4.1)
AUC (CI 95%)	0.849 (0.785-0.914)	0.756 (0.693-0.818)	0.710 (0.656-0.763)
p value	<0.001	<0.001	<0.001
Cut-off point	4.1	4.1	4.1
Se (CI 95%)	91.1 (80.7-96.1)	69.0 (58.5-77.9)	56.2 (47.6-64.4)
Sp (CI 95%)	79.4 (75.8-82.5)	80.3 (76.8-83.4)	81.5 (77.9-84.6)
PPV (CI 95%)	30.5 (24.1-37.9)	34.7 (27.9-42.2)	43.7 (36.4-51.3)
NPV (CI 95%)	98.9 (97.4-99.5)	94.5 (92.0-96.2)	87.9 (84.6-90.5)
PPR (CI 95%)	4.41 (3.68-5.29)	3.51 (2.81-4.38)	3.03 (2.39-3.85)
NPR (CI 95%)	0.11 (0.05-0.26)	0.39 (0.28-0.53)	0.54 (0.44-0.66)
Diagnostic accuracy	80.4 (77.1-83.4)	78.8 (75.5-81.8)	76.3 (72.9-79.5)
Odds ratio	39.22 (15.3-100.49)	9.11 (5.48-15.13)	5.64 (3.73-8.52)

Abbreviations: NEWS2: National early warning score 2; NEWS2-L: National early warning score 2-Lactate; Se: sensitivity; Sp: specificity; PPV: positive predictive value; NPV: negative predictive value; PPR: positive probability ratio; NPR: negative probability ratio.

## Discussion

In view of the results obtained, we can see that the NEWS2-L scale has a good ability to predict early clinical deterioration in patients with dyspnoea, significantly superior to the NEWS2 scale and similar to LA.

We also found that both analysed scales and LA lost their predictive capability over the long term. This loss of efficacy was most pronounced in the case of LA, which might be related to the fact that this biomarker increases considerably in situations of hypoxia and hypoperfusion(15) and that these situations are more relevant in early clinical deterioration but less important in long-term mortality. (16) This in turn would explain why LA showed such a similar efficacy to NEWS2-L in the first 48 hours in patients with dyspnoea, given that these patients are likely to present more situations of hypoxia than patients affected by other, non-respiratory syndromes.(17)

The results suggest that using NEWS2 type scales in combination with LA - in this case, the NEWS2-L scale - could be very worthwhile for predicting early clinical deterioration in patients with dyspnoea, helping prehospital emergency personnel and hospital critical care and emergency personnel in decision-making. Early warning scales have been attracting interest for some time because of their potential to stratify the risk of deterioration in complex patients and many studies have been conducted around them. In their systematic review on early warning scales, Patel *et al.* (18) concluded that very low values in these scales were capable of discriminating non-severity and very high values were able to predict significant early clinical deterioration.

Jo *et al.* (19) analysed whether associating lactate with the ViEWS early warning scale (ViEWS-L) could increase its predictive value. This scale demonstrated a greater predictive value for hospital mortality than ViEWS without LA (ViEWS-L 0.802 vs ViEWS 0.742, *p*=0.009), a superiority that was maintained at one, two, three and four weeks. These findings are in line with our own findings between the NEWS2-L vs NEWS2 scales, although we evaluated mortality at 48 hours and at seven and 30 days. Another study by Young *et al.*(20) carried out on haematology-oncology patients concluded that the application of an early warning scale (MEWS) in combination with LA significantly reduced the need to activate intensive care units due to patient deterioration, reinforcing the idea that associating an early warning scale with LA improves prognostic capacity.

A similar methodological study was conducted on this research, assessing another scale, called preNEWS2-L (Pre-Hospital National Early Warning Score 2 Lactate). It produced similar data in some aspects and an AUC-ROC of 0.91 (CI 95% 0.83-0.86) for mortality at 48 hours. However, that study referred specifically to the prehospital setting and included patients with all types of conditions, unlike the present study that only included patients with dyspnoea, so the obtained results are not directly comparable, although they do shore up the aforementioned idea of how associating LA with an early warning scale can improve its performance.(21)

Even though it is obvious that no scale can replace a suitable patient history and clinical examination by highly qualified personnel, many studies have concluded that the different early warning scales are a useful and efficient tool in predicting clinical deterioration among potentially serious patients, enabling a better clinical approach.(22-24) Karlotte *et al.,* in their systematic review(25) analysing the impact of early warning scales on nurses’ competence, concluded that these results were mainly beneficial in their professional practice, although in some cases they could produce contradictory outcomes. The increase in the use of these types of scale featuring biomarkers like LA could, as shown in this study, be of major help for nursing personnel who use these assessment tools.

This study has a number of limitations: firstly, there are many early warning systems, making it hard to select between them, although, as shown throughout the study, the NEWS2 scale has very high psychometric values today and is extensively used in both hospital and prehospital settings. Secondly, LA was used for its demonstrated clinical utility, but there are other analytical parameters such as blood gases, electrolytes, etc., which can today be obtained in the prehospital setting and could offer more data for clinical patient handling. Finally, this study focused on patients whose reason for assistance was dyspnoea and studied their mortality at 48 hours and at seven and 30 days after the values obtained at the time of prehospital care, but going forwards it would be interesting to have studies that assess the way the scale evolves using serial measurements in the hospital setting and appraising their efficacy in other conditions. It could also be very interesting to obtain and compare several early warning scales against each other and obtain other analytical values at the highest levels of lactate, studying the combinations that offer the most efficacy for predicting early clinical deterioration by groups of patients with different conditions.

In short, and after analysing the AUC-ROCs mentioned previously in the results, we can state that the NEWS2-L scale performed better than the NEWS2 scale and similar to LA in predicting mortality within the first 48 hours in patients seen for dyspnoea, and that the NEWS2-L scale beat LA as a predictor of mortality at seven and 30 days and was therefore superior overall. This scale can be useful in detecting patients likely to suffer significant clinical deterioration and more mortality from the time of their prehospital treatment, helping guide health professionals’ efforts to choose more or less intensive therapies with the aim of safeguarding the patient. Its regular use by emergency medical services could be considered to develop the most efficient and suitable response.

In the specific case of nurses, their role is fundamental to the correct implementation of the scale since they are the people who regularly collect the data needed to prepare it. Having a validated, well-performing scale like this facilitates their regular practice in the early detection of patients who are starting to deteriorate and can increase and guide therapeutic efforts. Correctly collecting the necessary variables, applying the scale and interpreting the result obtained, then consequently acting on it are also a challenge for nurses.
